# The Facebook Paradox: Effects of Facebooking on Individuals’ Social Relationships and Psychological Well-Being

**DOI:** 10.3389/fpsyg.2017.00087

**Published:** 2017-01-31

**Authors:** Xiaomeng Hu, Andrew Kim, Nicholas Siwek, David Wilder

**Affiliations:** Department of Psychology, Rutgers University–New Brunswick, PiscatawayNJ, USA

**Keywords:** Facebooking, online–oﬄine social contexts, social relationship satisfaction, psychological well-being

## Abstract

Research suggests that Facebooking can be both beneficial and detrimental for users’ psychological well-being. The current study attempts to reconcile these seemingly mixed and inconsistent findings by unpacking the specific effects of Facebooking on users’ online–oﬄine social relationship satisfaction and psychological well-being. Using structural equation modeling, pathways were examined between Facebook intensity, online–oﬄine social relationship satisfaction, perceived social support, social interaction anxiety, and psychological well-being. Personality differences on each of those paths were also assessed. Employing a sample of 342 American university students, results indicated that intensive Facebooking was positively associated with users’ psychological well-being through online social relationship satisfaction, and simultaneously negatively linked to users’ psychological well-being through oﬄine social relationship satisfaction. Multiple group analyses revealed that the linkage between perceived social support and psychological well-being was stronger for introverts than for extraverts. Our findings indicate that the benefits or detriments of Facebooking are contingent upon both personality characteristics and online–oﬄine social contexts.

## Introduction

### Facebook Psychology

Facebook is currently the largest online social network in the world and plays an active role in connecting people from distinct geographical regions and with diverse cultural backgrounds. According to the statistics released on its official website, its monthly active users currently exceed 1.79 billion and daily active users passed 1.18 billion as of September 2016 (Facebook, 2017). As Facebook use continues to be an integral part of people’s everyday life, enriching our understanding of the impact of Facebooking on its users will better inform researchers and the lay public alike about the psychological impact of using Facebook.

Facebook research to date has mainly examined five topics: descriptions of Facebook users, motivations for using Facebook, identity presentation, the effects of Facebook use on social interaction, and privacy concerns/information disclosure (Wilson et al., 2012). Psychological theories such as the dual factor model have been proposed to explain the primary drivers of Facebook use: belongingness and self-presentation (Nadkarni and Hofmann, 2012). Interestingly, current empirical evidence has yielded mixed and even contradictory results pertaining to its influence and implications for users’ social relationships and psychological well-being. On the one hand, studies have identified certain benefits and advantages of intensive Facebooking. For instance, intensity of Facebook use is positively linked to students’ life satisfaction, social trust, civic engagement, and political participation (Valenzuela et al., 2009), associated with three types of social capital: bonding (tightly knit, emotionally close relationships), bridging (broader identities and generalized reciprocity), and maintenance (connection to a previously inhabited community) (Ellison et al., 2007), and strongly predicts bridging social capital using longitudinal data (Steinfield et al., 2008). Furthermore, updating one’s Facebook status has been found to reduce loneliness by increasing users’ daily social connectedness (große Deters and Mehl, 2012). Smile intensity in Facebook photos predicts changes in life satisfaction over time (3.5 years later) (Seder and Oishi, 2012). Thus, studies seem to indicate that Facebooking can provide social psychological benefits for users’ social relationships and psychological well-being.

One the other hand, studies also reveal potential downsides of Facebook usage. For instance, the number of Facebook friends is negatively linked to self-esteem and academic adjustment in college (Kalpidou et al., 2011), positively related to romantic jealousy and relationship dissatisfaction (Elphinston and Noller, 2011), and greater Facebook use predicts declines in cognitive and affective well-being over time (Kross et al., 2013). Moreover, frequent Facebook interactions have also been found to be associated with greater stress directly and indirectly via a two-step pathway comprising of increased communication overload and reduced self-esteem (Chen and Lee, 2013). Additionally, “Facebook envy” may occur especially when people tend to share their most positive experiences to construct an appealing online persona. Indeed, evidence reveals that Facebook envy mediates the relation between Facebook surveillance and depression (Tandoc et al., 2015) and may pose a hidden threat to users’ life satisfaction (Krasnova et al., 2013). Even more puzzling, Facebook use is found to be simultaneously positively associated with both relatedness-need satisfaction and relatedness-need dissatisfaction (Sheldon et al., 2011). Overall, prior research suggests a paradoxical effect such that intensive Facebooking can be both helpful and harmful to users’ social engagements and psychological well-being.

In a similar vein, literature in Internet Psychology has highlighted an analogous phenomenon termed the “Internet Paradox” (Kraut et al., 1998). Internet use has been associated with increased levels of loneliness, depression, and decreased social support which may reduce social involvement and psychological well-being (Kraut et al., 2002). Other studies, however, have linked Internet use to decreased loneliness and depression along with increased perceived social support and self-esteem (Shaw and Gant, 2002). Borrowing from the “Internet Paradox,” we coin the term “Facebook Paradox” to describe the inconsistent and sometimes contradictory impact of Facebooking on users’ social relationships and psychological well-being.

Several hypotheses have been proposed to explain the interplay between online and oﬄine relationships. Previous research suggests that online relationships are much weaker than oﬄine ones (Vitak, 2008) and are also perceived as less close and less supportive (Mesch and Talmud, 2006). Psychologists contend that the growth of online connections may occur at the expense of oﬄine relationships (i.e., the displacement hypothesis) (Kraut et al., 1998). Alternatively, the social enhancement hypothesis posits that people who are more extroverted and with high self-esteem reap the benefits from Facebooking by augmenting their oﬄine popularity. Additionally, the social compensation hypothesis asserts that people who are more introverted and with low self-esteem compensate for their oﬄine inadequacy by controlling social interactions on Facebook (Zywica and Danowski, 2008). Although current findings are mixed and inconclusive with respect to the relations between online and oﬄine social contexts, distinguishing between online and oﬄine social relationships may help unpack the complex relations interplay between Facebooking and psychological well-being.

### Personality and Facebook Use

Personality characteristics can impact the way an individual behaves on the Internet. A growing body of literature has documented the relationships between personality traits and Internet use. For instance, according to the Oxford Handbook of Internet Psychology, some personality traits associated with Internet use include need for closure, need for cognition, locus of control, sensation seeking, risk taking, and the big five personality traits (Joinson, 2007). Moreover, research suggests that extraverted and unconscientious individuals engage in higher levels of social networking use (Wilson et al., 2010). Extraversion and neuroticism are linked to different preferences for Internet use such as information, leisure, or social services (Hamburger and Ben-Artzi, 2000). Finally, people high in neuroticism engage in more self-disclosing (actual, hidden, and ideal selves) and self-presentational behaviors (Seidman, 2013).

With regard to Facebook, prior research suggests that compared to non-Facebook users, Facebook users tend to be more extraverted and narcissistic but less conscientious and less socially lonely (Ryan and Xenos, 2011). Moreover, individuals with low self-esteem are more likely to actively engage in social compensatory friending (Lee et al., 2012) and feel safer disclosing themselves on Facebook, even though no direct social benefits are reaped (e.g., being more liked by their Facebook friends) (Forest and Wood, 2012). Additionally, people who have a preference for Facebook over Twitter see themselves as higher in sociability, extraversion, and neuroticism but lower in need for closure (Hughes et al., 2012). Other research findings indicate that the relationships between Facebook usage and personality traits are mixed and inconclusive (Gilbert and Barton, 2013). Thereby, more nuanced investigations are needed to further unpack the puzzling relationships between Facebooking and personality characteristics.

### The Present Research

The aims of the current study were threefold. First, we attempted to unravel the “Facebook Paradox” by examining possible pathways to explain how Facebooking affected individuals’ social relationship satisfaction and psychological well-being. Building upon prior research, we hypothesized that the intensity of Facebooking would be positively related to users’ online social relationship satisfaction (H1a) and negatively linked to their oﬄine social relationship satisfaction (H1b). We also hypothesized that the intensity of Facebooking would be positively related to users’ psychological well-being through their online social relationship satisfaction (H2a) and negatively linked with users’ psychological well-being via oﬄine social relationship satisfaction (H2b).

Second, we examined the roles that perceived social support and social interaction anxiety played in the abovementioned relationships. We predicted that perceived social support would mediate the relation between online social relationship satisfaction and psychological well-being (H3a), while social interaction anxiety would mediate the link between oﬄine social relationship satisfaction and psychological well-being (H3b).

Third, we explored the impact of certain personality characteristics. Extraversion and neuroticism have been shown to be two significant predictors of social networking use (Correa et al., 2010). Facebooking can help extraverted users maintain, reinforce, and expand their pre-existing social relationships (i.e., social enhancement hypotheses) (Zywica and Danowski, 2008), which in turn may increase their perceived social support and psychological well-being. Therefore, stronger positive relationships should exist among Facebooking, perceived social support, and psychological well-being for extraverts. Conversely, face-to-face social interactions can be somewhat difficult and challenging for introverts. Facebook, thereby, can serve as a psychologically comfortable platform to foster social relationships and promote self-disclosure (i.e., social compensation hypotheses) (Zywica and Danowski, 2008). Together, we hypothesized that relations among Facebooking, online social relationship satisfaction, perceived social support, social interaction anxiety, and psychological well-being would be stronger for introverts than for extroverts (H4a), whereas relations among Facebooking, oﬄine social relationship satisfaction, perceived social support, social interaction anxiety, and psychological well-being would be stronger for extraverts than for introverts (H4b).

Taken together, this research attempts to reconcile the reported inconsistencies and contradictions of prior findings by distinguishing context-specific social relationship satisfactions (i.e., online and oﬄine). To our knowledge, research has not yet examined the interrelations among these psychological constructs simultaneously. Structural equation modeling is a useful statistical technique to decompose complex interrelations. Therefore, path analyses were performed to test possible pathways among measured variables.

## Materials and Methods

### Participants

Four hundred and five college students at a large American public university participated in this study. Approval was obtained from the university’s institutional review board. Participants were invited to complete a set of online questionnaires. Participants who did not have a Facebook account or who did not provide full responses were excluded with the result that data from 342 participants were used for the final analyses. All participants were enrolled in introductory psychology, received one research credit for compensation, and were debriefed and thanked for their participation.

### Measures

#### Facebook Intensity

This construct was measured by six items on a 5-point Likert scale (1 = strongly disagree, 5 = strongly agree) that assessed the extent to which Facebook was used on a daily basis and how embedded Facebook was in the individual’s social life (Ellison et al., 2007) (e.g., “Facebook is part of my everyday activity”) (Cronbach’s alpha = 0.84).

#### Social Relationship Satisfaction Scale

This is a 5-point Likert scale (1 = very dissatisfied, 5 = very satisfied) which measured qualitatively and quantitatively a person’s social network of relationships. Two scales were created by adapting existing scales (Hendrick, 1988) with wording changed to reflect the context of either Facebook or oﬄine interactions (e.g., “How deep are the feelings you experience with your Facebook friends when you interact with them on Facebook”) (Cronbach’s alpha for online social relationship satisfaction = 0.82, and Cronbach’s alpha for oﬄine social relationship satisfaction = 0.86).

#### Perceived Social Support

This is a 16-item measure that asked participants to report how easy it was to obtain tangible help, advice, emotional support, and companionship on a 5-point Likert scale (1 = strongly disagree, 5 = strongly agree) (Cohen et al., 1985). A sample item is “There is someone I could turn to for advice about changing my job or finding a new one” (Cronbach’s alpha = 0.90).

#### Social Interaction Anxiety

This 19-item scale (SIAS) measured the extent to which individuals felt anxious in their social life on a 5-point Likert scale (1 = Not characteristic of me, 5 = Extremely characteristic of me) (Mattick and Clarke, 1998). Sample items are “I have difficulty talking with other people,” and “When mixing socially, I am uncomfortable” (Cronbach’s alpha = 0.93).

#### The Big Five Inventory

The 44-item Big Five personality test included scales that assessed the personality traits of Extraversion, Agreeableness, Conscientiousness, Neuroticism, and Openness (John et al., 2008). This measure was administered employing a 5-point Likert scale (1 = strongly disagree, 5 = strongly agree) (Cronbach’s alpha = 0.72).

#### Psychological Well-Being

This 5-item scale measured participants’ general life satisfaction on a 5-point Likert scale (1 = strongly disagree, 5 = strongly agree) (Diener et al., 1985). Sample items are “In most ways my life is close to my ideal” and “I am satisfied with my life” (Cronbach’s alpha = 0.86).

### Procedure

Information about the study was posted online to recruit participants. All questionnaires were administered using Qualtrics. Participants were presented an online consent form and then proceeded to complete a set of questionnaires. After participants submitted their responses, they were given a debriefing statement as well as granted one research credit.

### Data Analyses

**FIGURE 1:**
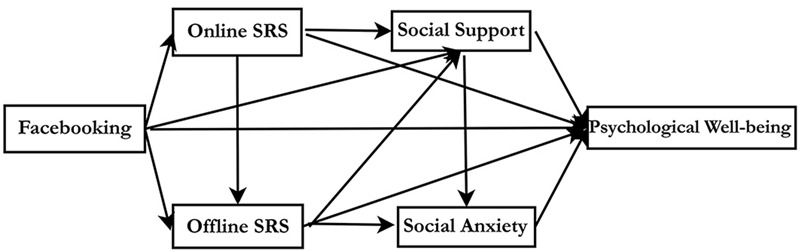
**The Hypothesized Path Model.** This figure shows Facebook intensity is both directly and indirectly positively associated with psychological well-being via online social relationship satisfaction and perceived social support, and is also directly and indirectly negatively linked to psychological well-being via oﬄine social relationship satisfaction and social interaction anxiety.

**FIGURE 2:**
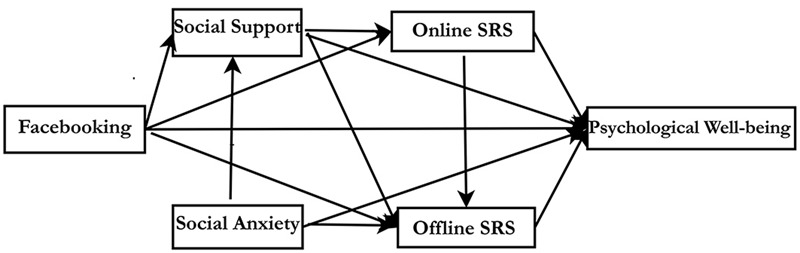
**The Alternative Hypothesized Path model.** This figure shows Facebook intensity is both directly and indirectly positively associated with psychological well-being via perceived social support and online social relationship satisfaction, and is also both directly and indirectly negatively linked to psychological well-being via oﬄine social relationship satisfaction but not social interaction anxiety.

To simultaneously examine the relationships among the measured psychological constructs of interest including overall model fit, direct effects, and indirect effects path analyses were performed using AMOS. A hypothesized model and an alternative model are shown in **Figures 1** and **2**.

The hypothesized path model was recursive and therefore identified (df = 5). Chi-square test, Comparative Fit Index (CFI), Tucker-Lewis index (TLI), and Root Mean Square Error of Approximation (RMSEA) were reported to assess the overall model fit. A non-significant chi-square (*p* > 0.05) (Barrett et al., 2007), TLI/CFI greater than 0.95 (Hu and Bentler, 1999), and RMSEA less than 0.05 (Hu and Bentler, 1999) indicate a good fitting model. Moreover, a bootstrapping technique (bias-corrected confidence intervals) was adopted to test the significance of the indirect effects. Inclusion of 0 between the confidence intervals would indicate significant mediation effects (Hayes and Scharkow, 2013). Finally, non-significant paths were eliminated to provide the final model.

An alternative model was also tested and compared. It may also be plausible that Facebook use could lead to increased or decreased psychological well-being through either perceived social support or social interaction anxiety first and then through either online or oﬄine social relationship satisfaction. Because those two models were not nested, model comparisons using Akaike Information Criterion (AIC), Bayesian Information Criterion (BIC) and Akaike’s Bayesian Information Criterion (ABIC) techniques were also reported. A decrease of 10 or more indicates a significantly improved model fit.

## Results

### Descriptive Statistics

There were no missing data for all measured variables. On average, participants reported moderate levels of Facebook intensity, perceived social support, social interaction anxiety, and psychological well-being. Participants were also more satisfied with their oﬄine social relationship than their online social relationship (*t* = 20.47, *p* < 0.001). The descriptive statistics are shown in **Table 1**, and the zero-order intercorrelations among the measured variables are shown in **Table 2**.

**Table 1:** Descriptive statistics.

	Demographics	*M* ±*SD*/*n* (%)
	Facebook intensity	3.33 ± 0.81
	Online social relationship satisfaction	3.30 ± 0.59
	Oﬄine social relationship satisfaction	4.09 ± 0.57
	Perceived social support	4.12 ± 0.63
	Social interaction anxiety	2.32 ± 0.75
	Psychological Well-being	3.25 ± 0.82
	Age	19.8 ± 2.2
Gender:		
	Male	98 (29%)
	Female	244 (71%)
Ethnicity		
	Caucasian/white	46.8
	Hispanic/Latino	10.2
	Asian/Asian American	14.9
	African American	5.8
	Native American/Alaskan native	3.5%
	Multiracial	2.6%

**Table 2:** Zero-order intercorrelation matrix.

	1	2	3	4	5	6
(1) Facebook Intensity	–	0.380^∗∗^	-0.022	0.097	0.011	0.120^∗^
(2) Online SRS^∗^		–	0.227^∗∗^	0.193^∗^	-0.150^∗^	0.214^∗∗^
(3) Oﬄine SRS^∗^			–	0.576^∗∗^	-0.393^∗∗^	0.404^∗∗^
(4) Perceived social support				–	-0.356^∗^	0.493^∗^
(5) Social interaction anxiety					–	-0.405^∗∗^
(6) Psychological well-being						–

*SRS, social relationship satisfaction. ^∗^Denotes correlation is significant at 0.05 level; ^∗∗^Denotes correlation is significant at 0.01 level*

### Testing Path Models

To first demonstrate that the six measured variables in our model are independent rather than overlapping with each other, we performed a CFA using AMOS. Treating each item within each scale as indicators and the six scales as latent variables, results indicated that the six-factor model fit better with our data [χ^2^(120) = 182.468, *p* < 0.001, RMSEA = 0.039, CFI = 0.984, TLI = 0.980] than alternative models such as the five-factor model [χ^2^(122) = 1040.853, *p* < 0.001, RMSEA = 0.136, CFI = 0.796, TLI = 0.754] and the one-factor model [χ^2^(152) = 2866.042, *p* < 0.001, RMSEA = 0.229, CFI = 0.383, TLI = 0.306]. These results suggest that all six unobserved variables: Facebook intensity, online social relationship satisfaction, oﬄine social relationship satisfaction, perceived social support, social interaction anxiety, and psychological well-being are distinctive psychological constructs.

**FIGURE 3:**
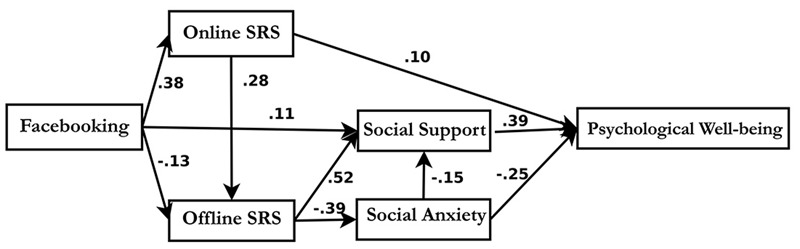
**The Best Fitting Trimmed-out Path Model.** This figure shows Facebook intensity is indirectly linked with psychological well-being via online social relationship satisfaction or perceived social support, and is also indirectly negatively linked to psychological well-being via oﬄine social relationship satisfaction or social interaction anxiety.

Path analyses were performed to investigate the relations among Facebooking, online and oﬄine social relationship satisfaction, perceived social support, social interaction anxiety, and psychological well-being. The model fit indices indicated that the data fit well with the covariance/correlation matrices [χ^2^(5) = 6.980, *p* < 0.001, RMSEA = 0.034, CFI = 0.995, TLI = 0.985] (see **Figure 3**).

#### Direct Effects

Facebooking both positively and negatively explained the variance of users’ psychological well-being. Specifically, the intensity of Facebook use had direct positive effects on users’ online social relationship satisfaction (Beta = 0.38, *B* = 0.277, *SE* = 0.037, *p* < 0.01), perceived social support (Beta = 0.109, *B* = 0.086, *SE* = 0.034, *p* < 0.05), and negative direct effects on users’ oﬄine social relationship satisfaction (Beta = -0.126, *B* = -0.089, *SE* = 0.040, *p* < 0.01). Furthermore, users’ online social relationship satisfaction was also positively linked to their oﬄine social relationship satisfaction (Beta = 0.275, *B* = 0.266, *SE* = 0.055, *p* < 0.01) and psychological well-being (Beta = 0.102, *B* = 0.142, *SE* = 0.063, *p* < 0.05), while user’s oﬄine social relationship satisfaction was positively linked to perceived social support (Beta = 0.518, *B* = 0.575, *SE* = 0.052, *p* < 0.01) and negatively associated with social interaction anxiety (Beta = -0.393, *B* = 0.515, *SE* = 0.065, *p* < 0.01). Additionally, social interaction anxiety was negatively related to perceived social support (Beta = -0.153, *B* = -0.130, *SE* = 0.040, *p* < 0.01) and psychological well-being (Beta = -0.254, *B* = -0.278, *SE* = 0.053, *p* < 0.01). Lastly, perceived social support had a strong positive impact on users’ psychological well-being (Beta = 0.384, *B* = 0.497, *SE* = 0.063, *p* < 0.01).

#### Indirect Effects

Facebooking also had indirect effects on users’ psychological well-being mediated by online social relationship satisfaction, perceived social support, oﬄine social relationship satisfaction, and social interaction anxiety. Bootstrapping mediation analyses suggested that the combination of those indirect effects was significant, 95% confidence interval (CI) = [0.035–0.145], *p* = 0.001. The intensity of Facebook use was also indirectly linked to perceived social support and social interaction anxiety. Mediation analyses indicated that this indirect effect was significant, CI = [0.056–0.734], *p* = 0.001. Moreover, oﬄine social relationship satisfaction had indirect effects on psychological well-being through perceived social support and social interaction anxiety. Those joint mediation effects were significant, CI = [0.002–0.388], *p* = 0.001.

**FIGURE 4:**
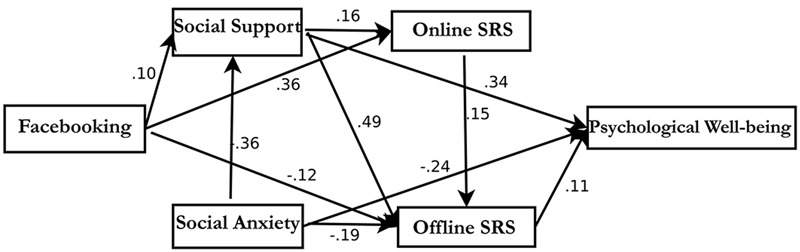
**The Alternative Trimmed-out Path Model.** This figure shows Facebook intensity is indirectly linked with psychological well-being via perceived social support but not via online social relationship satisfaction, and is also indirectly negatively linked to psychological well-being via oﬄine social relationship satisfaction but not via social interaction anxiety.

The alternative model did not demonstrate improved fit to the data compared with the final path model [χ^2^(5) = 125.281, *p* < 0.001, RMSEA = 0.266, CFI = 0.705, TLI = 0.115] (see **Figure 4**). AIC, BCC, and BIC indices indicated that the final model was improved relative to other competing models (see **Table 2**).

### Personality Differences

Multiple group analyses were performed to determine whether there were any personality effects on the model level and the path level. Results indicated that the unconstrained model and fully constrained model were not different from each other neither between extraverts and introverts [Δχ^2^(5) = 6.896, *p* = 0.228] nor between neurotics and non-neurotics [Δχ^2^(6) = 4.598, *p* = 0.596]. At the path level the link between perceived social support and psychological well-being was stronger for introverts than for extraverts [Δχ^2^(1) = 4.851, *p* < 0.05]. No other path yielded significant differences between extraverts and introverts or between neurotics and non-neurotics.

## Discussion

### Summary of Main Findings

The present study aimed to explore how Facebooking was associated with users’ psychological well-being through their online and oﬄine social relationship satisfaction, perceived social support, and social interaction anxiety. We also looked at whether these effects were moderated by personality characteristics. Our results indicate that Facebooking is both positively and negatively linked with users’ psychological well being, partially mediated by perceived social support and social interaction anxiety (H1, H2, and H3 were supported). Taken together, our findings provide further evidence for the “Facebook Paradox” in which Facebook usage can be predictably helpful or harmful to well-being. Our research demonstrates that distinguishing users’ online and oﬄine social contexts and taking personality traits into account can help tease apart the “Facebook Paradox.”

### Personality Effects

The link between perceived social support and psychological well-being was stronger for introverts than for extraverts (H4a was partially supported). This suggests that the psychological well-being of introverted users depends more heavily upon perceived social support. However, we did not find other personality differences especially between online and oﬄine social contexts (H4b was not supported). Thereby, our findings did not fully support the social enhancement and social compensation hypotheses (Zywica and Danowski, 2008). Future research could further address whether Facebook can serve as an enhancer for extraverted users and a compensatory tool for introverted users to promote psychological well-being.

### Theoretical and Practical Implications

Our results demonstrate that intensive Facebooking has both direct and indirect benefits and detriments to users’ social functioning and psychological well-being via different routes. The specific effects of Facebooking on users’ psychological well-being are context-dependent and mediated by perceived social support and social interaction anxiety. This research also suggests that online and oﬄine social contexts can complement and reinforce each other. On the one hand, fostering online social relationships on Facebook can expand users’ social network, accumulate social capital, prompt users to engage actively in social groups, and promote self-presentation. All of these outcomes may benefit oﬄine social relationships via obtaining richer information, facilitating deeper social interactions, and boosting self-efficacy. On the other hand, reinforcing oﬄine social relationships enables individuals to obtain intimacy, interpersonal trust, and strong social ties. This, in turn, may help Facebook users maintain more meaningful and closer social relationships in online social contexts.

### Contributions

The research reported here provides empirical evidence that addresses some of the inconsistent findings on the effects of Facebook usage on psychological well-being (“Facebook Paradox”). Our study also identifies the mediating roles that perceived social support and social interaction anxiety play in the relationships between Facebooking and psychological well-being.

We also introduced the construct of online social relationship satisfaction as a mediator of the impact of Facebooking on psychological well-being. This construct may provide helpful insights to better our understanding of the mixed relationships between Facebooking and well-being that have been reported in previous research.

### Limitations and Future Directions

The cross-sectional nature of our data limits the ability to make causal claims for the relationships among the tested psychological constructs. This research also exclusively relied on self-report measures which may reflect biases such as social desirability. However, other research has shown that self-report data did not significantly differ from users’ observed information on their Facebook profiles (Gosling et al., 2007). Additionally, our sample only included university students which may reduce the generalizability of the findings. Nevertheless, college students are an appropriate group to study Facebook psychology as they have been raised with Facebook and can be viewed as “digital natives.”

## Conclusion

Taken together, our findings indicate that Facebooking can be both beneficial and detrimental for users’ psychological well-being through online or oﬄine social relationship satisfaction. Specially, Facebooking is positively associated with users’ psychological well-being through their online social relationship satisfaction or perceived social support, and negatively linked to users’ psychological well-being through oﬄine social relationship satisfaction, perceived social support, and social interaction anxiety. Furthermore, the link between perceived social support and psychological well-being appears to be stronger for introverts than for extraverts. Addressing the Facebook Paradox, the present study suggests that whether Facebooking is helpful or harmful to users’ social relationships and psychological well-being is contingent upon both personality characteristics and online-oﬄine social contexts.

## Ethics Statement

This study was exempt from the Institutional Review Board for the Protection of Human Subjects at Rutgers University, the State University of New Jersey because it involved less than minimal risk for the participants.

## Author Contributions

XH: Designed and carried out the study, collected and analyzed data, wrote up the manuscript; AK: Assisted with data collection; NS: Assisted with data collection; DW: Refined the research idea, supervised the study throughout, and revised the manuscript.

## Conflict of Interest Statement

The authors declare that the research was conducted in the absence of any commercial or financial relationships that could be construed as a potential conflict of interest.
